# The Synaptic Interactions of Alcohol and the Endogenous Cannabinoid System

**DOI:** 10.35946/arcr.v42.1.03

**Published:** 2022-01-27

**Authors:** Sarah A. Wolfe, Valentina Vozella, Marisa Roberto

**Affiliations:** Department of Molecular Medicine, Scripps Research Institute, La Jolla, California

**Keywords:** endocannabinoid, alcohol use disorder, alcohol, synaptic, cannabis use disorder, cannabinoid receptor, cannabis, neurobiology

## Abstract

**PURPOSE:**

A growing body of evidence has implicated the endocannabinoid (eCB) system in the acute, chronic, and withdrawal effects of alcohol/ethanol on synaptic function. These eCB-mediated synaptic effects may contribute to the development of alcohol use disorder (AUD). Alcohol exposure causes neurobiological alterations similar to those elicited by chronic cannabinoid (CB) exposure. Like alcohol, cannabinoids alter many central processes, such as cognition, locomotion, synaptic transmission, and neurotransmitter release. There is a strong need to elucidate the effects of ethanol on the eCB system in different brain regions to understand the role of eCB signaling in AUD.

**SEARCH METHODS:**

For the scope of this review, preclinical studies were identified through queries of the PubMed database.

**SEARCH RESULTS:**

This search yielded 459 articles. Clinical studies and papers irrelevant to the topic of this review were excluded.

**DISCUSSION AND CONCLUSIONS:**

The endocannabinoid system includes, but is not limited to, cannabinoid receptors 1 (CB_1_), among the most abundantly expressed neuronal receptors in the brain; cannabinoid receptors 2 (CB_2_); and endogenously formed CB_1_ ligands, including arachidonoylethanolamide (AEA; anandamide), and 2-arachidonoylglycerol (2-AG). The development of specific CB_1_ agonists, such as WIN 55,212-2 (WIN), and antagonists, such as SR 141716A (rimonabant), provide powerful pharmacological tools for eCB research. Alcohol exposure has brain region–specific effects on the eCB system, including altering the synthesis of endocannabinoids (e.g., AEA, 2-AG), the synthesis of their precursors, and the density and coupling efficacy of CB_1_. These alcohol-induced alterations of the eCB system have subsequent effects on synaptic function including neuronal excitability and postsynaptic conductance. This review will provide a comprehensive evaluation of the current literature on the synaptic interactions of alcohol exposure and eCB signaling systems, with an emphasis on molecular and physiological synaptic effects of alcohol on the eCB system. A limited volume of studies has focused on the underlying interactions of alcohol and the eCB system at the synaptic level in the brain. Thus, the data on synaptic interactions are sparse, and future research addressing these interactions is much needed.

Alcohol use disorder (AUD) is a chronic, relapsing brain disorder, characterized by a compromised ability to control alcohol use despite adverse occupational, social, or health consequences. Results from a 2019 National Survey on Drug Use and Health found that 5% of individuals over age 12 had AUD, affecting 14.5 million people in the United States. Alcohol and cannabis products are a common polydrug combination.^1^ Use of cannabinoids and alcohol alters many central processes, such as cognition, locomotion, and neuropeptide signaling.^2^ Cannabis use is associated with the development and maintenance of AUD,^3^ and individuals with cannabis use disorder (CUD) have an increased likelihood for development of comorbid AUD and double the risk for long-term problem drinking.^3^ The risks associated with polysubstance use with alcohol and cannabis are greater than those associated with use of either drug alone.^3^ Decriminalization has increased the availability and use of cannabis products^4^ and polysubstance use, raising multiple social and health concerns.^5^,^6^

The high prevalence of comorbid AUD and CUD may be explained, in part, through findings indicating that alcohol and cannabis serve as a substitute for one another, as both have overall depressing effects on the central nervous system (CNS) and produce feelings of intoxication and euphoria.^7^–^9^ Additionally, chronic ethanol administration in animal models causes neurobiological alterations similar to those elicited by chronic cannabinoid exposure,^10^ and shared physiological and biochemical mechanisms may contribute to their combined use. Although cannabis and alcohol have varying targets and effects, both have been shown to interact through the endogenous cannabinoid (endocannabinoid [eCB]) system.^11^ Ethanol changes the eCB system by altering the synthesis of eCBs, the synthesis of their precursors, and the density and coupling efficacy of cannabinoid receptor 1 (CB_1_), a G protein–coupled receptor and a major receptor of the eCB system.^12^–^14^ Furthermore, eCBs acting at CB_1_ can modulate alcohol consumption in rats by affecting the activity of brain reward systems^15^–^17^ and the function of the eCB system in AUD.^18^–^20^

Few studies have combined these two lines of research to fully understand the neurobiological substrates and synaptic interactions of alcohol and eCBs, or the therapeutic potential of targeting the eCB system for treating AUD. Therefore, this review provides an overview of the literature concerning how alcohol administration dysregulates eCB signaling and modulates eCB-mediated synaptic function. An emphasis is given to brain regions highly implicated in AUD and existing pharmacotherapies that target the eCB system and influence alcohol-perturbed synaptic functions. Additionally, a discussion of suggested future directions is provided to assist in addressing the lack of insights on the mechanisms and specific circuits at work in the synaptic interactions between alcohol and the eCB system.

The current literature indicates an urgent need for mechanistic studies to shed light on how perturbation of the brain eCB system contributes to development of AUD.

## Method

For the scope of this review, preclinical studies were identified through queries of the PubMed database. The initial PubMed searches were undertaken in March 2021, with a final updated search date of June 2021, using the following terms: (endocannabinoids OR cannabinoid OR CB1 OR CB2 OR anandamide OR 2-arachidonoylglycerol OR FAAH OR MAGL OR DAGL OR NAPE-PLD) AND (chronic OR acute OR alcohol OR ethanol OR withdrawal) AND (hippocampus OR amygdala OR nucleus accumbens OR ventral tegmental area OR striatum OR cerebellum OR cortex OR prefrontal cortex) AND (synaptic OR synapse). This search yielded 459 articles. All articles containing relevant information and supporting the topics discussed in this review were included. These articles include research and findings related to the endocannabinoid pathway and acute, chronic, and withdrawal alcohol interactions in all brain regions and in specific regard to interactions pertaining to synaptic structure, function, and adaptations. Articles were excluded if they pertained only to clinical research, behavioral research, or findings outside of the brain and unrelated to synaptic/neuronal function. To support the topics covered, this review includes additional citations that did not appear in the search but that were considered relevant.

## Results

### The Endogenous Cannabinoid System: An Overview

The cannabinoid receptors were identified in the late 1980s, 2 decades after the discovery of the bioactive and psychoactive effects of delta-9-tetrahydrocannabinol (THC).^21^,^22^ THC is one of 500 different compounds found in the plant *Cannabis sativa*, 85 of which are known cannabinoids (CBs).^23^ THC is the compound mainly responsible for the psychotropic effects of cannabis and elicits its psychoactive effects through binding specific G protein–coupled receptors (GPCRs), termed cannabinoid receptors.^21^,^22^ Two types of cannabinoid receptors were discovered via molecular cloning, the cannabinoid receptor type 1 (CB_1_)^24^ and the cannabinoid receptor type 2 (CB_2_).^25^–^27^ CB_1_ is the most abundant GPCR in the mammalian brain, where it is primarily found on presynaptic terminals. CB_1_ is also expressed at lower, but physiologically relevant, levels in most peripheral tissues.^20^,^28^ CB_2_ is abundant in the peripheral systems, and predominantly expressed in cells of the immune and hematopoietic systems. CB_2_ is also present in the CNS, but at much lower concentrations compared to CB_1_.^25^,^26^,^29^,^30^ Discovering the role of CB_2_ in the CNS is still ongoing.^26^,^31^ Both CB_1_ and CB_2_ are primarily positively coupled to G_i_/G_o_ proteins, and generally signal through inhibition of adenylate cyclase (AC), inhibition of calcium channels, and activation of potassium channels, thus regulating numerous cellular processes.^19^,^20^,^28^,^32^

The discovery of these specific CB receptors led to the isolation of their endogenously formed ligands, including two lipid-derived principal eCBs, arachidonoylethanolamide (anandamide [AEA]) and 2-arachidonoylglycerol (2-AG).^33^–^36^ AEA is a partial agonist with high affinity for CB_1_, whereas 2-AG is a full agonist with a lower affinity for CB_1_.^37^ Other GPCRs and other targets also recognize CBs and related endogenous lipids; however, their role is less well understood.^38^,^39^ For instance, both AEA and 2-AG bind to and activate the postsynaptic transient receptor potential vanilloid 1 and are agonists for several subtypes of the peroxisome proliferator-activated receptor family.^40^ AEA and 2-AG are synthesized on demand from membrane phospholipid precursors. These eCBs are arachidonic acid derivatives, biosynthesized through a combination of several pathways.^19^,^41^ AEA is mainly synthesized by the enzyme *N*-acyl phosphatidylethanolamine phospholipase D (NAPE-PLD),^42^ but other enzymes important for synthesis include glycerophosphodiester phosphodiesterase 1 (GDE1), abhydrolase domain containing 4 (ABHD4) and the protein tyrosine phosphatase, non-receptor type 22 (PTPN22).^19^,^41^ AEA is primarily catabolized by fatty acid amide hydrolase (FAAH), a serine hydrolase,^43^ and 2-AG is synthesized from diacylglycerol (DAG) through the catalytic activity of diacylglycerol lipase alpha (DAGL-alpha) and DAGL-beta.^29^,^44^ Catabolism of 2-AG occurs primarily by monoacylglycerol lipase (MAGL),^45^ but other relevant contributors include abhydrolase domain containing 6 and 12 (ABHD6 and ABHD12).^46^

The eCB system is essential to many cellular processes and is implicated in signaling cascades that modulate synaptic processes such as calcium signaling, synaptic transmission, and neurotransmitter release.^19^,^28^,^41^ In neurons, eCBs are synthesized and released postsynaptically, on demand, and in response to synaptic activity/membrane depolarization through calcium-dependent processes. The eCBs signal in a retrograde manner by traversing the synapse to bind their targets (i.e., CB_1_) on the presynaptic membrane.

**Figure 1:**
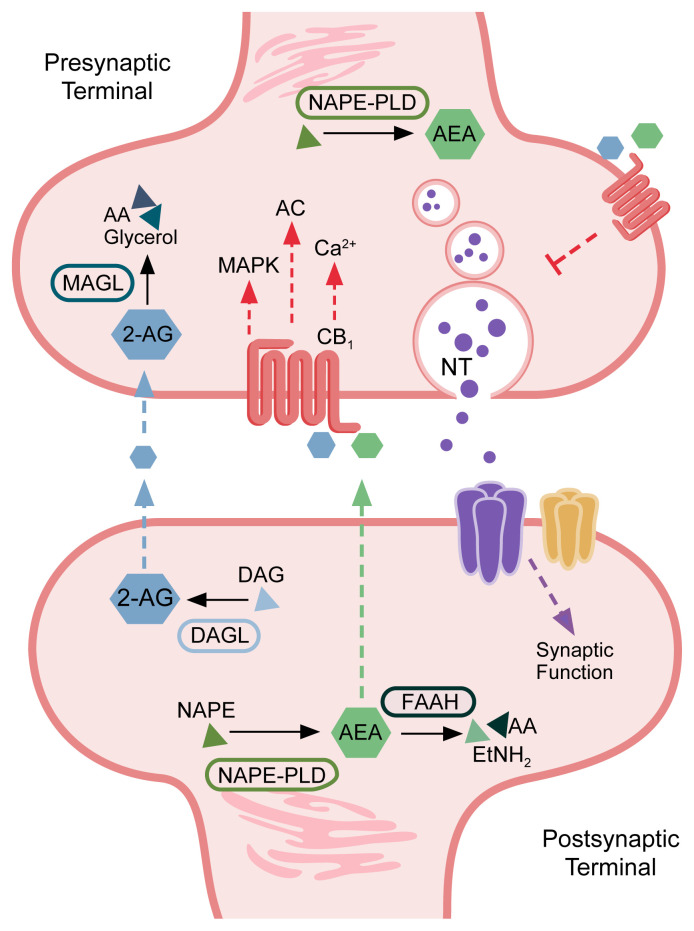
Summary schematic of endocannabinoid signaling in the synapse A simplified description of the subcellular distribution of components of the endocannabinoid pathway is shown. Components include the major enzymes involved in regulating endocannabinoid levels (fatty acid amide hydrolase [FAAH], *N*-acyl phosphatidylethanolamine [NAPE], NAPE-specific phospholipase D [NAPE-PLD], monoacylglycerol lipase [MAGL], and diacylglycerol lipase-alpha [DAGL-alpha]); major endocannabinoids (anandamide [AEA], 2-arachidonylglycerol [2-AG]); lipid precursors and metabolites (arachidonic acid [AA], 2-acylglycerol [AG], diacylglycerol [DAG], and ethanolamine [EtNH_2_]); cannabinoid receptor 1 (CB_1_); neurotransmitter (NT); and major signaling cascade mediators downstream of CB_1_ activity (mitogen-activated protein kinases [MAPK], adenylate cyclase [AC], and calcium [Ca^2+^] signaling). Endocannabinoids signal in a retrograde manner to activate presynaptic CB_1_, which mediates signaling mechanisms that influence synaptic transmission and neurotransmitter release.

The eCBs activate CB_1_ on both gamma-aminobutyric acid-ergic (GABAergic)^47^–^49^ and glutamatergic terminals.^50^ This presynaptic CB_1_ activation provides feedback inhibition via the suppression of neurotransmitter release^51^,^52^ in both inhibitory^53^–^55^ and excitatory synapses.^56^ However, alternative mechanisms for eCB release and CB_1_ activation do occur; for example, the activity of metabotropic glutamate receptor subtype 5 (mGluR5)^57^ and *N*-methyl-D-aspartate (NMDA) receptors^58^,^59^ can stimulate eCB production and subsequent release to bind and activate presynaptic CB_1_ receptors.^60^–^64^ The eCB system therefore serves as a critical mechanism for modulating neuronal activity. CB_1_ activation can lead to short- and long-term forms of plasticity, such as depolarization-induced suppression of inhibition/excitation and a form of synaptic long-term depression.^65^,^66^ Long-term depression is characterized by a reduction in the efficacy of synapses in an activity-dependent manner.^65^,^66^ The induction of these different forms of plasticity is probably linked to the activation of postsynaptic neurons that modulate concentration of eCBs at the synapse, the timing of CB_1_ activation, and downstream effectors.^67^ CB_2_ is involved in a long-lasting cell-type–specific form of plasticity that triggers neuronal hyperpolarization.^68^ The eCB system functions are reviewed by Lu and Anderson,^29^ Basavarajappa,^32^ and Basavarajappa et al.^41^ Figure 1 provides a summary schematic of synaptic eCB signaling.

### The Endocannabinoid Pathway and Alcohol Interactions

There is a high degree of comorbidity between AUD and CUD, which indicates a functional link between alcohol and cannabis.^18^ Synergistic effects also have been observed in rodents. For instance, co-administration of ethanol and cannabinoids has additive effects on some behaviors such as sleep,^69^ cognitive, psychomotor, and attention deficits.^70^ Additionally, alcohol and cannabis use might cause cross-tolerance,^18^,^71^ and acute tolerance of alcohol is thought to be mediated through the eCB system.^72^ Synergistic behaviors are reviewed by Pava and Woodward,^18^ Basavarajappa et al.,^19^ Kunos,^20^ and Henderson-Redmond et al.^73^

Although the focus of this review is the synaptic mechanisms of eCBs and alcohol, a brief description of the behavioral implications is provided for context throughout. The eCB system has emerged as a promising druggable target for the development of therapeutic options to treat AUD. Pharmacological modulation of the eCB system by CB receptor agonists, antagonists, eCB-degrading enzyme inhibitors, or anandamide transporter inhibitors alters the alcohol-related behaviors in rodents. Rats treated with CB_1_ antagonist SR 141716A (rimonabant), or its analog surinabant (SR 147778), showed reduced alcohol consumption and motivation to consume alcohol in various drinking models.^74^–^79^ CB_1_ agonists WIN 55,212-2 (WIN) and CP 55,940 increased ethanol consumption and preference in mice and rats.^80^,^81^ Activation of CB_2_ signaling using the agonist JWH133 seems to reduce both alcohol- and food-rewarding behaviors.^82^ The expression and function of CB_1_ receptors and FAAH are altered in AUD,^83^,^84^ and pretreatment with the FAAH inhibitor URB597 reduced alcohol intake and preference after acute withdrawal through a CB_1_-mediated mechanism.^85^ However, URB597 administration increased operant ethanol self-administration in rats,^84^ whereas AEA transport blocker AM404 had efficacy in reducing ethanol self-administration in rodent models.^86^ The discrepancy between the effects of the FAAH inhibitor URB597^84^ and the AEA transport blocker in models of alcohol self-administration might be due to the mechanism of action of AM404,^86^ which does not involve the CB_1_ receptor, given that the administration of CB_1_ antagonists or agonists does not affect alcohol self-administration.^86^ Interestingly, recent findings from Soria-Gomez et al. have shown that the activation of CB_1_ at different subcellular locations (plasma membrane vs. mitochondria) within the same circuit is associated with opposite behavioral outcomes.^87^ This observation might shed light on why alcohol often has discrepant effects on the activation or inhibition of the eCB system and vice versa.^87^

Ethanol and cannabinoids induce neurophysiological consequences through their interaction with specific substrates (i.e., receptors and enzymes). Although cannabinoids primarily modulate synaptic neurotransmission via the eCB system, ethanol interacts with a variety of different molecular substrates that affect a diverse range of neurochemical processes. The eCB system plays a critical role in mediating the effects of ethanol in the brain, contributing to ethanol-induced biochemical, genetic, electrophysiological, and behavioral consequences. This suggests that eCB signaling contributes to the underlying neuropathology that drives AUD.^18^ Despite this strong brain implication, the synaptic mechanisms of alcohol and eCB signaling are still not fully investigated, and some brain regions involved in the addiction cycle are relatively unexplored. Additionally, alcohol paradigms vary across studies, and acute, chronic, and withdrawal exposures are not fully characterized within specific brain regions. Therefore, the following discussion of the current literature on synaptic eCB and alcohol interactions is divided into two main sections: (1) acute alcohol exposure and (2) chronic alcohol exposure and withdrawal. Each section is subdivided by brain region—where data are available—including the hippocampus, amygdala, prefrontal cortex, basolateral amygdala (BLA), nucleus accumbens (NAc), ventral tegmental area (VTA), striatum, and cerebellum.

### Acute Alcohol Exposure and eCB System Interactions

Acute alcohol exposure produces intoxicating effects by acting on the CNS, both at low and high concentrations (1–100 mM) in preclinical animal or cell culture experiments and nontolerant humans.^88^ Acute concentrations of ethanol can directly interact with several molecules and have specific effects on different brain regions.^89^ Ethanol has rapid acute effects on the function of proteins involved in excitatory and inhibitory synaptic transmission.^88^ Some of these effects are mediated by eCB signaling and subsequent alterations in neurotransmission and synaptic activity. However, the eCB system is complex, and ethanol-induced effects are brain region–specific and sensitive to the exposure methodology used. Therefore, discrepancies between studies occur, possibly because of differences in methodology, tissue/cell culture, and ethanol exposure paradigm.

#### Hippocampus

Acute alcohol exposure is known to affect hippocampal function and to impact contextual and episodic memory by altering neuronal processes.^90^ In general, acute alcohol exposure consistently decreases eCB (AEA, 2-AG) levels as measured directly in tissue of the striatum, hippocampus, prefrontal cortex, amygdala, and cerebellum.^91^–^93^ The decreases in eCBs observed are not due to increased metabolism by FAAH activity and therefore are not mediated by metabolic activity and degradation of eCBs.^91^ Furthermore, FAAH activity in the hippocampus was transiently decreased 45 minutes post intraperitoneal (IP) injection of ethanol (4 g/kg).^91^ However, as stated earlier, discrepancies between studies occur, possibly due to methodology, differences in tissues/cell cultures, and ethanol exposure paradigm. For example, in contrast to the above studies, acute alcohol exposure in hippocampal neurons increased both AEA and 2-AG levels via a calcium-dependent mechanism and subsequently inhibited presynaptic glutamate release.^94^ Acute ethanol exposure did not alter CB_1_ presynaptic expression but did enhance both AEA and 2-AG.^94^ Ethanol-induced alterations in CB receptor activity and eCB levels affect the eCB system and may lead to disruptions in synaptic function. Ethanol decreases the frequencies, but not amplitude, of spontaneous miniature excitatory postsynaptic currents (mEPSCs), suggesting inhibition of vesicular glutamate release and suppression of synaptic functions.^94^ These studies overall demonstrate the complex role of eCB signaling in regulating ethanol-induced effects in the hippocampus.

Cannabinoids and acute alcohol exposure alter synaptic transmission in the hippocampus through the eCB system. Specifically, cannabinoid exposure inhibited glutamatergic synaptic transmission in hippocampal cultures^95^ and inhibited calcium currents in cell cultures.^96^ In rat hippocampal cultures, the cannabimimetic WIN inhibited N- and P/Q-type calcium channels through the CB_1_ receptor whereas the nonpsychoactive enantiomer, WIN 55,212-3, was not effective. Maximal inhibition by the nonclassical cannabinoid agonist CP 55,940 was similar to that seen with maximal concentrations of WIN.^97^

#### Amygdala

The extended amygdala represents a macrostructure composed of several basal forebrain structures: the bed nucleus of the stria terminalis, central medial amygdala (CeA), and a transition zone in the posterior part of the medial NAc (i.e., posterior shell).^98^–^100^ Key elements of the extended amygdala include not only neurotransmitters associated with the positive reinforcing effects of substances such as alcohol, opioids, cocaine, and amphetamines, but also major components of the brain stress systems associated with the negative reinforcement of drug dependence.^100^–^102^ CB_1_ in part regulates the effects of alcohol in CeA neurons, and activation of CB_1_ attenuates the alcohol effect on the CeA’s gamma-aminobutyric acid (GABA) system.^11^ Acute application of ethanol in an ex vivo CeA brain slice induced presynaptic facilitation of GABAergic signaling on rat CeA neurons via increased GABA release.^103^–^105^ This ethanol-induced, evoked, and spontaneous GABA release was blocked by CB_1_ activation via the agonist WIN.^54^,^55^ Similarly, superfusion of WIN prevented subsequent ethanol effects on GABAergic transmission. The application of CB_1_ antagonists rimonabant and AM251 alone augmented GABAergic responses, revealing a tonic eCB activity that decreased inhibitory transmission in CeA via a presynaptic CB_1_ mechanism. The intracellular calcium chelator BAPTA abolished the ability of AM251 to augment GABA responses, demonstrating the eCB-driven nature and postsynaptic origin of the tonic CB_1_-dependent control of GABA release. Notably, the ethanol-induced facilitation of GABA release was additive to CB_1_ blockade, ruling out participation of CB_1_ in the action of acute ethanol.^54^,^55^ These studies on both evoked and spontaneous GABA transmission point to an important role of CB_1_ in the CeA, in which the eCBs tonically regulate neuronal activity and suggest a potent mechanism for modulating CeA tone during challenge with ethanol.^54^

CB_1_ activation is known to decrease glutamate release in many brain areas, including the CeA, of male rodents.^51^,^106^ Glutamatergic transmission also was investigated in the CeA of Wistar and Marchigian Sardinian alcohol-preferring (msP) rats.^107^ Notably, msP rats display enhanced anxiety, stress, and alcohol drinking, simulating the alcohol-dependent phenotype. Findings indicate that acute ethanol application decreases evoked excitatory postsynaptic potential amplitudes in rat CeA. WIN decreased glutamatergic responses via presynaptic mechanisms in male rats only, and combined application of WIN and acute ethanol exposure resulted in strain-specific effects in females.^107^ No tonic CB_1_ signaling at glutamatergic synapses in the CeA of any groups, and no interactions with ethanol were observed. Collectively, these observations demonstrate sex strain–specific differences in ethanol and endocannabinoid effects on CeA glutamatergic signaling.^107^

#### Basolateral amygdala

The eCB system in the BLA plays a role in gating stress and anxiety responses by modulating GABA and glutamate transmission.^108^,^109^ CB_1_ is highly expressed in cholecystokinin-positive GABAergic interneurons^110^,^111^ and at lower levels in glutamatergic pyramidal cells.^111^ A wide body of work has demonstrated that CB_1_ activity decreases GABAergic transmission in the BLA.^110^,^112^–^114^ GABAergic transmission in the BLA is increased by acute ethanol exposure in naïve rats via both presynaptic and postsynaptic mechanisms. Although CB_1_ activation impairs ethanol’s facilitation of GABAergic transmission, ethanol’s presynaptic site of action is likely independent of CB_1_, given that acute ethanol application further increases GABA release in the presence of a CB_1_ antagonist.^115^ CB_1_ antagonism with rimonabant or chronic pretreatment with CB_1_ agonist WIN attenuates acute alcohol-induced inhibition of neuronal firing in the BLA.^116^ Further evidence shows that eCBs are either not released or cannot activate CB_1_ receptors in the presence of ethanol, resulting in GABAergic transmission under conditions when they would normally be suppressed.^117^ Interestingly, ethanol prevented depolarization-induced suppression of inhibition even when the postsynaptic neuron was loaded with AEA during the experiment, suggesting that increasing the eCBs available for release could not overcome the ethanol effect.^117^

#### Nucleus accumbens

The NAc mediates emotional and reward-related stimuli by integrating signals from the limbic system.^101^,^118^,^119^ In the NAc, acute ethanol altered eCB system components, which may affect NAc function. Acute alcohol IP administration (15% ethanol, 4 g/kg) increased AEA and CB_1_ binding in rat NAc^120^ and in immature mouse hippocampus and cortex.^121^ Therefore, acute alcohol enables eCB synthesis and release.^94^,^116^ Self-administration of ethanol (10% for 30 minutes) by rats acutely increased 2-AG interstitial levels in the NAc shell during ethanol exposure with no concurrent alteration in AEA, as measured by in vivo microdialysis. Interestingly, the relative change in dialysate 2-AG levels was positively correlated with the amount of ethanol consumed.^122^

In the NAc, acute ethanol exposure enhances dopamine release, which can be inhibited by blockade or genetic ablation of CB_1_, suggesting that acute alcohol exposure facilitates the dopaminergic system via the eCB system.^123^ In awake, freely moving rats, acute ethanol treatment (IP injection) induced a dose-dependent release of dopamine in the dopaminergic projection area of the NAc.^124^ This ethanol-induced release of dopamine was exacerbated in alcohol-preferring rats when compared to alcohol-avoiding rats.^125^ With CB_1_ activation (via THC or WIN), dopamine release was elicited in the rat NAc shell similarly to that induced by alcohol,^126^ and CB_1_ activity induced an increase in spontaneous firing due to inhibition of GABAergic inputs onto projections of dopaminergic neurons to the NAc (see the VTA section below for detail).^127^–^129^ Modulation of the dopamine system in the NAc is complex, and activation of CB_1_ on prefrontal cortex glutamatergic terminals in the NAc reduces glutamatergic transmission and consequently dopamine. This may limit the rewarding effects of acute alcohol exposure.^130^

#### Ventral tegmental area

The VTA is known to mediate the positive reinforcement effect of alcohol. Dopaminergic neurotransmission in the VTA was identified as a key mechanism for the establishment and maintenance of alcohol intake.^131^ Similar to the NAc, acute alcohol exposure increased the firing rate of VTA dopaminergic neurons in a CB_1_-dependent manner.^17^ CB_1_ is not expressed on dopaminergic neurons in the VTA; therefore, the eCB-induced increase in dopamine release in the VTA is mediated by CB_1_ activity on inhibitory GABAergic interneurons. This results in disinhibition of dopaminergic neurons in the VTA and increased dopamine release in the NAc.^128^,^129^

#### Striatum

The striatum is implicated in habit formation and motivation or goal-directed actions, and acute alcohol exposure disrupts the stability of striatal neuronal circuits.^132^ In the striatum, the physiological effects of acute ethanol exposure appear to oppose, or are antagonized by, eCB signaling mechanisms. In the rat dorsomedial striatum, acute alcohol exposure inhibited eCB release from medium spiny neurons, preventing lasting disinhibition. This effect was found to be independent of eCB synthesis and CB_1_ activity. In the rat dorsomedial striatum, release of eCBs from medium spiny neurons is associated with disinhibition of these neurons for an extended period of time and decreased synaptic long-term depression. This long-lasting disinhibition can be blocked independently of CB_1_ activation or synthesis of eCBs by pretreatment with alcohol. Acute ethanol treatment prevents the long-lasting disinhibition induced by the CB_1_ agonist WIN in rat striatum. These data suggest that the eCB system is involved in the physiological response to acute alcohol intoxication.^132^

#### Cerebellum

Cerebellum function can be affected by alcohol, causing disruptions in locomotion, balance, and executive functions. Acute alcohol exposure impairs cerebellar function by altering gamma-aminobutyric acid type A (GABA_A_) receptor-mediated neurotransmission.^133^ Ethanol induces presynaptic GABA release onto cerebellar Purkinje neurons through a pathway that is dependent on protein kinase A (PKA) and that releases calcium from internal stores independent of eCB synthesis.^134^ In contrast, activation of CB_1_ in Purkinje neurons inhibits the ethanol-induced GABA release from presynaptic terminals and the frequency of inhibitory postsynaptic currents (IPSCs). This blockade of ethanol-induced IPSC frequency is mediated by the PKA pathway, through G protein (G_i_)-mediated inhibition of PKA produced by activation of CB_1_.^135^ Notably, CB_1_ activation by WIN also blocked ethanol from increasing spontaneous GABA release onto the interneuron–Purkinje cell synapses in the cerebellum.^135^

#### Summary

The above studies (summarized in Table 1) indicate that acute alcohol exposure profoundly affects the eCB system, including expression and function of eCB signaling components that subsequently impact neuronal function and synaptic transmission. It is also evident that acute ethanol exposure differentially affects the eCB system depending on brain region and alcohol administration method. Further difficulties in elucidating alcohol and the eCB system interactions arise from the complexity of the eCB pathway due to its retrograde signaling on both GABAergic and glutamatergic synapses.^20^,^29^,^32^,^41^ Additionally, factors such as the state of tissue or cells under study (ex vivo, in vivo, or in vitro) or the species (mice or rats) may affect results.^18^ Although alcohol-related behavioral studies implicate the importance of the eCB system, the underlying effects induced by acute ethanol exposure on the synaptic interactions between alcohol and the endogenous cannabinoid system are not well understood.

### The eCB System in Chronic Alcohol Exposure and Alcohol Withdrawal

Chronic ethanol exposure induces many neuroadaptive changes in the CNS involving both GABAergic and glutamatergic synaptic transmission. Long-term ethanol exposure results in both tolerance and dependence. Tolerance presents as a decreased behavioral response to ethanol and decreased intoxication. Dependence is described by symptomatology elicited during and following ethanol withdrawal, including anxiety, hyperalgesia, dysphoria, susceptibility to seizures, and disrupted sleep states.^88^ Both chronic ethanol and cannabinoid exposure produce similar adaptations in eCB signaling.^10^ Cross-tolerance with alcohol and cannabis also is consistent with changes in CB_1_ expression.^18^ Preclinical studies using different chronic ethanol treatment models have consistently observed reduced CB_1_ expression or function in a variety of rodent brain regions^136^–^139^ and in alcohol-preferring rats.^140^–^142^ However, as with acute exposure to alcohol, effects of chronic alcohol exposure may vary depending on exposure paradigm and may be brain region–specific. In humans, chronic heavy drinking (defined as greater than six drinks per day, where a standard drink contains ~ 10g of ethanol) is linked to reduced CB_1_ receptor availability and binding in numerous brain regions that persist after prolonged abstinence or withdrawal, and amount of alcohol intake is negatively correlated with years of misuse.^137^,^143^ Chronic dysregulation of the eCB system suggests a mechanism underlying the negative affect associated with AUD.^20^ Although the effects of alcohol withdrawal on the eCB pathway are not well known, alcohol withdrawal in some cases recovers the effects induced by chronic alcohol exposure on components of the eCB system.^120^,^136^,^144^–^147^

#### Hippocampus

Chronic ethanol exposure induced structural and functional changes in the hippocampus.^118^,^148^,^149^ This region is also highly sensitive to the damaging effects of chronic alcohol use.^90^ Multiple studies demonstrate that chronic alcohol exposure and withdrawal dysregulate the hippocampal eCB system. Regional dysfunction was identified in CB_1_, indicated by reduced relative CB_1_ binding, in the hippocampus and caudate-putamen of rats exposed to alcohol via liquid diet for 7 days.^120^ A 7-day alcohol paradigm reduced WIN sensitivity and induced altered monoamine synthesis in the locus coeruleus, hippocampus, and striatum.^150^ Additionally, genetic deletion of CB_1_ impaired the neuroadaptations of NMDA and GABA_A_ receptors in the cerebral cortex and hippocampus induced by chronic ethanol treatment, indicating that the eCB system plays a critical role in alcohol dependence.^151^

Alcohol-dependent rats (52 days of forced access) were found to have reduced CB_1_ gene expression (measured via *Cnr1* messenger RNA [mRNA] levels) in the hippocampus, hypothalamus, and striatum.^141^ Similarly, chronic intermittent ethanol (CIE) exposure via oral intubation (55 days of forced access followed by 2 days of withdrawal) in rats reduced *Cnr1* expression and CB_1_ levels in the hippocampus.^139^ In alcohol-preferring msP rats, *Cnr1* expression was greater in several brain regions including the frontoparietal cortex, caudate-putamen, and hippocampus, although this was reversed following alcohol self-administration.^140^ Sardinian alcohol-preferring (sP) rats, compared to alcohol–non-preferring rats, display greater CB_1_ density, *Cnr1* levels, and eCB levels in the cerebral cortex, hippocampus, and striatum. Reduced FAAH expression also was observed in the hippocampus of sP rats.^147^ Consistent with these findings, 12 weeks of CIE vapor reduced *Cnr1* and CB_1_ levels in the rat lateral habenula, while enhancing levels of the eCB-related mRNA and/or proteins, DAGL-beta, NAPE-PLD mRNA (*napepld*), and MAGL.^152^ In contrast, no change in CB_1_ receptor binding and mRNA levels occurred in the hippocampus, cerebral cortex, or motor and limbic structures in a chronic ethanol intake model (7% liquid diet for 15 days).^153^

The eCB system’s role in alcohol withdrawal in the hippocampus is not well understood, and studies are variable. The dysfunction in CB_1_ identified by Ceccarini et al. was reversed after 2 weeks of abstinence from alcohol.^120^ However, another study identified lasting effects on eCBs; even with 40 days of withdrawal, alcohol-dependent rats retained enhanced AEA and 2-AG levels in the hippocampus.^139^ Despite this molecular evidence, synaptic studies on the functional consequences of the changes observed in eCBs are lacking.

#### Prefrontal cortex

Chronic alcohol exposure affects the structure and function of the prefrontal cortex, causing deficits in executive control, decision-making, and risk management.^154^ As observed in the hippocampus, chronic alcohol exposure induces alterations in NMDA and GABA_A_ receptor expression in wildtype mice, but not in CB_1_-depleted mice, indicating that the eCB system plays a critical role in alcohol dependence.^151^ Additionally, in situ hybridization in msP rats identified that *Cnr1* expression is greater in the frontoparietal cortex; this was reversed following alcohol self-administration.^140^ However, no change in CB_1_ receptor binding and mRNA levels occurred in the cerebral cortex with chronic ethanol intake (7% liquid diet for 15 days).^155^

Acute application of the CB_1_ agonist WIN enhanced the amplitude of the period of depolarization (up states) in slice cultures of the prefrontal cortex but not in slices that underwent 10 days of chronic ethanol treatment followed by 4 days of withdrawal. Chronic ethanol followed by 4 days of withdrawal blunted WIN inhibition of evoked GABA inhibitory postsynaptic currents (IPSCs) in layer II/III of the pyramidal neurons but not in layer V/VI. WIN inhibited the amplitude of spontaneous GABA IPSCs in both layers and this effect was not altered by ethanol treatment.^144^ Some studies indicate that alcohol withdrawal may lessen the effects of eCB system alterations induced by chronic alcohol exposure. CIE exposure increased *Cnr1* expression in the medial prefrontal cingulate cortex, and alcohol withdrawal recovers the effects of chronic exposure to control levels in rats.^145^ Acute alcohol withdrawal also produced reduction in gene expression of components of the eCB system and reduced 2-AG content in the medial prefrontal cortex of male rats, but not in female rats.^146^

#### Amygdala

In the amygdala, eCB signaling is compromised in alcohol-dependent animal models. Chronic alcohol intake in rats (7% liquid diet for 15 days) induced a decrease in both 2-AG and AEA in the midbrain and an increase in AEA in the limbic forebrain, but no change occurred in CB_1_ receptor binding and mRNA levels in limbic structures.^136^,^153^,^155^ A chronic ethanol liquid diet (10% ethanol, continuous access for 15 days; or intermittent access for 5 days/week for 3 weeks) followed by acute withdrawal (6 or 24 hours) significantly altered gene expression for a variety of components of the amygdala’s eCB system. Reductions in FAAH, MAGL, CB_1_, CB_2_, and GPR55 mRNA were observed, with alteration in MAGL and CB receptor–associated mRNA being more pronounced with intermittent alcohol exposure.^156^ In the CeA, an alcohol self-administration paradigm decreased 2-AG levels in dependent rats, and MAGL inhibitors increased alcohol consumption.^157^ In baseline CeA dialysate, AEA and 2-AG levels decreased in ethanol-dependent rats with further decrements during 12-hour withdrawal. Subsequent ethanol consumption restored 2-AG dialysate content to baseline levels.^157^,^158^ MsP rats also displayed higher FAAH activity and decreased AEA levels in the CeA as measured by microdialysis.^142^

GABAergic dysregulation in the CeA is a hallmark of the transition to alcohol dependence in animal models.^101^ A study by Varodayan and colleagues reported that activation of CB_1_ via WIN decreased the frequency of spontaneous and miniature CeA GABA_A_ receptor-mediated IPSCs, which could be blocked by CB_1_ antagonism.^55^ Two weeks of CIE vapor significantly blunted this effect of WIN. Chronic ethanol exposure abolished tonic CB_1_ influence on vesicular GABA release, indicating that CB_1_ function in the CeA is impaired by chronic ethanol exposure.^55^ Therefore, decreased CB_1_ activity is likely a factor that contributes to the dysregulated (enhanced) GABA transmission in the CeA with chronic alcohol exposure.^55^ Altered eCB function may contribute to the dependence-associated disruptions in glutamate and GABA transmission in the CeA.^11^,^103^ These findings indicate that eCB signaling is compromised in the amygdala of ethanol-dependent rats, contributing to an allostatic shift toward maintenance of ethanol intake through negative reinforcement.^34^,^54^,^158^

#### Basolateral amygdala

Chronic ethanol exposure and withdrawal alter synaptic transmission in the BLA.^114^,^116^,^159^–^161^ Emotional processing is affected by the actions of CB_1_ on GABA and glutamate neurotransmission in the BLA.^108^–^110^,^112^–^114^,^162^,^163^ Decreased CB_1_ and increased AEA levels were observed in the BLA with a 10-day CIE vapor paradigm.^164^ Additionally, ethanol exposure caused a dose-dependent inhibition of glutamatergic synaptic activity via a presynaptic mechanism that was occluded by CB_1_ antagonists rimonabant and AM251. Importantly, this acute ethanol inhibition was attenuated following CIE.^164^ Withdrawal produced a reduction in the gene expression of *Cnr1* and the protein levels of DAGL-alpha, MAGL, and AEA levels in the BLA of male rats, but not female rats.^146^ In naïve rats, WIN application decreased GABA release, which was prevented by CB_1_ antagonist AM251. AM251 increased GABA release via a postsynaptic, calcium-dependent mechanism. This retrograde tonic CB_1_ signaling was reduced in rats exposed to 2 weeks of CIE, suggesting impaired eCB signaling. These results indicate that CB_1_ has a critical role in regulating BLA GABAergic transmission, which is dysregulated with chronic ethanol exposure.^115^

#### Ventral tegmental area

Few studies have investigated chronic alcohol exposure in the VTA. However, one study conducted in mice identified that VTA GABA_A_ receptor inhibition in dopaminergic neurons was regulated through presynaptic actions of eCBs. The same study showed that withdrawal from CIE vapor exposure increased eCB-mediated inhibition on GABA synapses of VTA dopamine neurons.^165^ Withdrawal was shown to decrease sensitivity to WIN and enhance sensitivity to AM251, suggesting that GABA_A_ inhibition of dopamine neurons in the VTA is regulated by presynaptic eCB activity and that withdrawal increases eCB-mediated inhibition.^165^

#### Striatum

In the rat striatum, chronic alcohol treatment is associated with dysregulation of the eCB system, specifically with a decrease in *Cnr1* mRNA levels.^140^,^141^ Similar to the cortex, hippocampus, and cerebellum, a 72-hour ethanol vapor inhalation paradigm decreased CB_1_ receptor density and CB_1_ activation in mouse striatum. These effects were recovered after 24 hours of withdrawal from ethanol, suggesting that these eCB neuroadaptations may play a role in development of tolerance and dependence.^136^,^147^ In sP rats, greater CB_1_ density, CB_1_ mRNA, CB_1_-mediated G protein coupling, and eCB levels were observed in the striatum. Alcohol intake (homecage two-bottle free-choice regimen with unlimited access for 24 hours/day for 70 consecutive days) in sP rats reduced CB_1_-mediated G protein coupling, which was reversed by rimonabant administration, and increased eCBs in the striatum, associating the eCB system with higher alcohol preferences.^147^ Studies in humans also identified altered eCB signaling components. Human postmortem tissue from patients with AUD has decreased CB_1_ expression, decreased FAAH expression and activity, and increased AEA levels, all specifically identified in the ventral striatum.^166^

Additionally, synaptic plasticity may be influenced by ethanol and mediated via the eCB system. CIE vapor in mice abolished CB_1_-mediated long-term depression in the mouse dorsolateral striatum and increased 2-AG.^167^ These results suggest that chronic ethanol exposure causes neuroadaptations in the striatum that may contribute to the progression of AUD in humans and alcohol dependence in animals.^167^

#### Cerebellum

Analogous to acute exposure, chronic alcohol exposure disrupts cerebellar function through GABA_A_ and eCB mechanisms.^133^ As in the striatum, chronic ethanol exposure decreased CB_1_ receptor density and activity in the mouse cerebellum, which was reversed with withdrawal.^136^ In cultured cerebellar granular neurons and cultured neuronal cells (human neuroblastoma SK-N-SH), 72 hours of ethanol exposure increased the synthesis of AEA and 2-AG through calcium activation of phospholipase A2 and subsequently increased NAPE-PLD activity in cultured cells.^19^,^138^,^168^ Additionally, in mouse synaptic plasma membrane, chronic alcohol exposure decreased the function and expression of CB_1_.^138^,^169^,^170^ Similarly, chronic alcohol intake induced an increase in AEA levels and a decrease in components of AEA transport and FAAH in cultured cerebellar neurons.^171^

#### Summary

Overall, these data (summarized in Table 2) indicate that chronic alcohol exposure compromises CB_1_ and eCB pathways, and alcohol withdrawal may ameliorate these effects. The chronic alcohol-induced molecular changes in the eCB system—including the synthesis of eCBs and the expression of CB_1_ and catabolizing enzymes—have a profound impact on neuronal function and synaptic transmission in multiple brain regions.^13^,^155^ These effects with alcohol withdrawal may be due to a compensatory effect to regulate neurotransmission and counteract neuroadaptations induced with chronic alcohol exposure. The strong association of polydrug use with alcohol and cannabis products presents the possibility of self-medicating for AUD with cannabis and developing CUD.^18^,^172^,^173^ Further research on the eCB pathways may facilitate the modulation of the eCB system as a target for future AUD treatment.

## General Summary and Future Directions

There is clear evidence that the eCB system plays a critical role in the acute effects of alcohol on synaptic functions, and that neuroadaptations occur with chronic alcohol exposure and withdrawal in eCB signaling. The eCB system orchestrates a complex signaling mechanism. Ethanol- and/or withdrawal-induced molecular alterations in the eCB system impact neuronal functions and synaptic transmission in a brain region–specific manner. A variety of studies have demonstrated the potential beneficial effects of several pharmacological approaches for treating AUD by modulating the eCB system.^84^,^156^,^157^,^174^ A growing number of CB_1_ and CB_2_ agonists and antagonists, FAAH and MAGL inhibitors, as well as NAPE-PLD and DAGL inhibitors have been developed in the past 2 decades. However, determining how ethanol exposure affects eCB metabolizing enzymes at the synaptic level requires further research and will provide invaluable insight to guide our understanding of the pathophysiology of alcohol-induced synaptic changes. Specifically, FAAH and MAGL inhibitors have been proven efficacious in ameliorating the negative affect in preclinical models of AUD.^157^,^174^–^177^ However, more research is needed to understand how these compounds affect synaptic transmission.

Many studies have identified the importance of eCB signaling in mediating behavioral responses to alcohol exposure and withdrawal; however, the underlying neuronal mechanism is not well characterized. Unfortunately, the current literature is limited and lacks the consistency (length of ethanol exposure, time of measurements, neurochemicals measured, etc.) across brain regions that is necessary for a more comprehensive understanding of the synaptic interactions of the eCB system and alcohol. However, a few studies that are consistent indicate strong themes within brain regions. For instance, a variety of chronic ethanol exposure paradigms in the hippocampus consistently indicated a reduction in CB_1_ function, assessed via CB_1_ gene expression,^139^,^141^ binding,^120^ and WIN sensitivity,^150^ in most studies and in multiple rat strains.^140^,^147^ In studies where a similar methodology is used, such as in the amygdala, strong and consistent evidence identified the role of CB_1_ in the effects of acute alcohol exposure.^11^,^54^,^55^ CB1 was found to attenuate the acute ethanol-induced facilitation of GABAergic signaling in the CeA.^54^,^55^ Combined, these studies identified an important role of the eCB system in modulating CeA signaling during alcohol exposure. However, in many cases, studies and research are insufficient to draw a detailed and comprehensive consensus of the synaptic role of the eCB system within different alcohol stages and brain regions. From the review of the literature, some recurring limitations emerged from the available studies. Therefore, the following are suggested as potential and important avenues of future research to address this gap in knowledge: (1) an emphasis on the synaptic protein landscape and synaptic function related to eCB signaling and alcohol interactions; (2) a focus on brain region specificity, given that different alterations in the eCB system are observed with alcohol exposure depending on brain region; (3) more consistent alcohol administration methodologies to control for differences in the eCB system that appear to be sensitive to different alcohol administration paradigms; (4) more research on the role that eCB signaling plays in alcohol withdrawal, particularly because very few studies have addressed this in terms of synaptic function; and (5) more research to address the lack of information concerning female animals and sex-specific differences as well as age-related effects.

Understanding the underlying mechanisms of alcohol and cannabinoid interaction in the different brain regions affected by AUD is still ongoing. Elucidating the role played by the eCB system in the alterations that occur in neural signaling and synaptic function after ethanol exposure and withdrawal may provide targets for developing pharmacotherapies for AUD. Additional mechanistic and physiological studies are needed to better understand how perturbations of the brain’s eCB system may contribute to development of AUD.

## Figures and Tables

**Table 1:** Acute Ethanol Exposure and ECB System Interaction, by Brain Region and Study

Brain Region and Study	Ethanol Exposure	System	Species	Measure	Effect	Drug	Synaptic Activity	Effect
**Hippocampus**								
Ferrer et al. (2007)^91^	4 g/kg, IP	Tissue	Wistar rats	AEA, 2-AG	Decrease			
Rubio et al. (2009);^92^ Rubio et al. (2007)^93^	24h liquid diet	Tissue	Sprague-Dawley rats	AEA, 2-AG	Decrease			
Ferrer et al. (2007)^91^	4 g/kg, IP	Tissue	Wistar rats	FAAH activity	Decrease			
Basavarajappa et al. (2008)^94^	30 and/or 60 min, 50 mM	Cultured neurons	C57BL/6J mice	AEA, 2-AG CB_1_ expression Presynaptic glutamate release	Increase No change Inhibition			
**Amygdala**								
Roberto et al. (2004);^103^ Roberto et al. (2004);^104^ Roberto et al. (2003)^105^	5–10 min, 44 mM	Brain slice	Sprague-Dawley rats	GABA transmission	Increase			
Roberto et al. (2010);^54^ Varodayan et al. (2016)^55^	5–10 min, 44 mM	Brain slice	Sprague-Dawley rats			WIN	Evoked and spontaneous GABA responses	Blockade
Roberto et al. (2010);^54^ Varodayan et al. (2016)^55^	5–10 min, 44 mM	Brain slice	Sprague-Dawley rats			Rimonabant, AM251	Evoked and spontaneous GABAergic responses	Increase
Kirson et al. (2018)^107^	10–15 min, 44 mM	Brain slice	Wistar and msP rats	Glutamatergic transmission	Decrease	WIN AM251	Evoked glutamatergic response (evoked EPSCs)	Further inhibition (males) and blockade of ethanol effect (Wistar females) with WIN No change with AM251
**Basolateral amygdala**								
Varodayan et al. (2017)^115^	5–10 min, 44 mM	Brain slice	Sprague-Dawley rats	GABAergic transmission	Increase	WIN AM251	Spontaneous GABAergic transmission (GABA release)	Reduction with WIN Increase with AM251
Perra et al. (2008)^116^	0.25–2.0g/kg, IV	Brain slice	Sprague-Dawley rats			Rimonabant, WIN chronic pretreatment	Inhibition of neuronal firing by ethanol	Reduction
**Nucleus accumbens**								
Ceccarini et al. (2013)^120^	4 g/kg, IP	Tissue	Wistar rats	AEA, CB_1_ binding	Increase			
Caillé et al. (2007)^122^	30 min self-administration	Dialysate	Wistar rats	2-AG AEA	Increase No change			
Hungund et al. (2003)^123^	1.5 g/kg, IP, 20–280 min	Dialysate	Mice	Dopamine release	Increase	CB_1_ knockout, Rimonabant	Dopamine release with ethanol	Inhibition
Di Chiara et al. (1988)^124^	0.25–2.5 g/kg, IP	Dialysate	Sprague-Dawley rats	Dopamine release	Increase			
**Ventral tegmental area**								
Perra et al. (2005)^17^	0.5 g/kg, IV	Brain slice	Sprague-Dawley rats	Dopaminergic neurons firing	Increase			
**Striatum**								
Clarke et al. (2009)^132^	20 min, 20–50 mM	Brain slice	Wistar rats	eCB release	Inhibition and prevention of long-lasting neuronal disinhibition			
**Cerebellum**								
Kelm et al. (2007)^134^	5 min, 50–100 mM	Brain slice	Sprague-Dawley rats	Presynaptic GABA release	Increase			
Kelm et al. (2008)^135^	5 min, 50–100 mM	Brain slice	Sprague-Dawley rats			WIN	Presynaptic GABA release (sIPSCs)	Inhibition

*Note:* 2-AG, 2-arachidonoylglycerol; AEA, arachidonoylethanolamide (anandamide); CB_1_, cannabinoid receptor 1; eCB, endocannabinoid; EPSCs, excitatory postsynaptic currents; FAAH, fatty acid amide hydrolase; GABA, gamma-aminobutyric acid; IP, intraperitoneal; IV, intravenous; sIPSCs, spontaneous inhibitory postsynaptic currents; WIN, WIN 55,212-2.

**Table 2:** Chronic Ethanol Exposure, Withdrawal, and ECB System Interaction, by Brain Region

Brain Region and Study	Ethanol Exposure	System	Species	Measure	Effect	Drug	Synaptic Activity	Effect
**Hippocampus**								
Ceccarini et al. (2013)^120^	7 days liquid diet (7% v/v)	Tissue	Wistar rats	CB_1_ binding	Reduction			
Ceccarini et al. (2013)^120^	7 days liquid diet (7% v/v) + 2 weeks abstinence	Tissue	Wistar rats	CB_1_ binding	Recovery			
Ortiz et al. (2004)^141^	52 days forced access	Tissue	Wistar rats	CB_1_ gene expression	Reduction			
Mitrirattanakul et al. (2007)^139^	55 days oral intubation (6 g/kg daily) + 2 days withdrawal	Tissue	Sprague-Dawley rats	CB_1_ gene expression, CB_1_ protein	Reduction			
Cippitelli et al. (2005)^140^	30 min daily sessions on a fixed ratio 1 schedule of reinforcement self-administration	Tissue	msP rats	CB_1_ gene expression	Reduction			
González et al. (2002)^153^	15 days liquid diet (7% v/v)	Tissue	Wistar rats	CB_1_ binding and gene expression	No change			
Mitrirattanakul et al. (2007)^139^	55 days oral intubation (6 g/kg daily) + 40 days withdrawal	Tissue	Sprague-Dawley rats	CB_1_ gene expression, CB_1_ protein, AEA, 2-AG	Increase			
**Prefrontal cortex**								
Cippitelli et al. (2005)^140^	18 days self-administration (10% v/v in 30 min daily sessions on a fixed ratio 1 schedule reinforcement)	Brain slice	msP rats (and Wistar rats)	CB_1_ gene expression	Reduction			
González et al. (2002)^155^	15 days liquid diet (7% v/v)	Tissue	Wistar rats	CB_1_ binding and gene expression	No change			
Pava et al. (2014)^144^	4 days withdrawal after 10 days chronic ethanol	Slice cultures	C57BL6/J mice			WIN	Spontaneous GABA transmission	No change
Rimondini et al. (2002)^145^	7 weeks intermittent alcohol (17 h/day)	Tissue	Wistar rats	CB_1_ gene expression	Increase			
Rimondini et al. (2002)^145^	3 weeks after 7 weeks of intermittent alcohol	Tissue	Wistar rats	CB_1_ gene expression	Recovery			
Henricks et al. (2017)^146^	Acute (1–4 days) withdrawal after 6 weeks chronic intermittent alcohol vapor	Tissue	Wistar rats	2-AG	Reduction			
**Amygdala**								
González et al. (2002)^153^,^155^	15 days liquid diet (7% v/v)	Tissue	Wistar rats	AEA CB_1_ binding and gene expression	Increase No change			
Serrano et al. (2012)^156^	Withdrawal after 5 days per week for 3 weeks	Tissue	Wistar rats	CB_1_, MAGL gene expression	Reduction			
Serrano et al. (2018)^157^	30 min on a fixed ratio 1 schedule self-administration	Dialysate	Wistar dependent rats	2-AG	Decrease			
Serrano et al. (2018);^157^ Chevaleyre et al. (2006)^158^	12 h withdrawal	Dialysate	Wistar dependent rats	AEA, 2-AG	Decrease			
Varodayan et al. (2016)^55^	2–3 weeks CIE vapor for 14 h a day	Brain slice	Sprague-Dawley rats			WIN, AM251	Spontaneous GABA transmission (GABA release)	CIE blunts WIN effect
**Basolateral amygdala**								
Robinson et al. (2016)^164^	10 days CIE vapor	Tissue; Brain slice	Sprague-Dawley rats	AEA CB_1_	Increase Decrease		Glutamatergic transmission	Inhibition
Robinson et al. (2016)^164^						Rimonabant, AM251	Glutamatergic transmission	Reverted ethanol-induced inhibition
Henricks et al. (2017)^146^	Acute (1–4 days) withdrawal after 6 weeks chronic intermittent alcohol vapor	Tissue	Wistar rats	AEA CB_1_, DAGL, MAGL gene expression	Reduction Reduction			
Varodayan et al. (2017)^115^	2–3 weeks CIE vapor for 14 h a day	Brain slice	Sprague-Dawley rats			WIN, AM251	Spontaneous GABA transmission	CIE reduced WIN- and AM251-mediated effect
**Ventral tegmental area**								
Harlan et al. (2018)^165^	3 weeks withdrawal from CIE vapor	Brain slice	C57BL6/J mice	sIPSC frequency	Reduced	WIN, AM251	eCB-mediated GABA_A_ inhibition (evoked IPSCs)	Increase
**Striatum**								
Cippitelli et al., (2005);^140^ Ortiz et al. (2004)^141^	30-min daily sessions on a fixed ratio 1 schedule of reinforcement self-administration	Tissue	Wistar rats	CB_1_ gene expression	Decrease			
Vinod et al. (2006)^136^	72 h ethanol vapor (10–16 mg/l)	Tissue	Swiss Webster mice	CB_1_ density and activation	Decrease			
Vinod et al. (2006)^136^	72 h ethanol vapor (10–16 mg/l) + 24 h withdrawal	Tissue	Swiss Webster mice	CB_1_ density and activation	Recovery			
Vinod et al. (2012)^147^	70 days of two-bottle choice (24 h access/day)	Tissue	sP rats	CB_1_-mediated G protein coupling eCB	Reduction Increase	Rimonabant	CB_1_-mediated G protein coupling	Reversed
DePoy et al. (2013)^167^	2 weeks intermittent ethanol (16 h/day for 4 days per week)	Brain slice	C57BL6/J mice	2-AG	Increase		CB_1_-mediated long-term depression	Abolition
**Cerebellum**								
Vinod et al. (2006)^136^	72 h ethanol vapor (10–16 mg/l)	Tissue	Swiss Webster mice	CB_1_ density and activation	Decrease			
Vinod et al. (2006)^136^	72 h ethanol vapor (10–16 mg/l) + 24 h withdrawal	Tissue	Swiss Webster mice	CB_1_ density and activation	Recovery			
Basavarajappa et al. (1999);^138^ Basavarajappa et al. (2000)^168^	72 h ethanol (100 mM)	Cultured cerebellar granular primary neurons and SK-N-SH (human cell line)	Sprague-Dawley rats	AEA, 2-AG synthesis	Increase	Rimonabant	Ethanol induced 2-AG synthesis	Inhibited
Basavarajappa et al. (1999);^138^ Basavarajappa et al. (2000)^168^	72 h ethanol (100–150 mM)	Cultured cerebellar granular primary neurons and SK-N-SH (human cell line)	Sprague-Dawley rats	NAPE-PLD activity	Increase			
Basavarajappa et al. (2003)^171^	72 h ethanol (100–150 mM)	Cultured cerebellar granular primary neurons	Sprague-Dawley rats	AEA transport FAAH activity	Decrease Decrease	Rimonabant	AEA transport	No change

*Note:* 2-AG, 2-arachidonoylglycerol; AEA, arachidonoylethanolamide (anandamide); CB_1_, cannabinoid receptor 1; CIE, chronic intermittent ethanol; FAAH, fatty acid amide hydrolase; GABA, gamma-aminobutyric acid; GABA_A_, gamma-aminobutyric acid type A receptor; MAGL, monoacylglycerol lipase; NAPE-PLD, *N*-acyl phosphatidylethanolamine–specific phospholipase D; sIPSC, spontaneous inhibitory postsynaptic current; WIN, WIN 55,212-2.
